# Left Ventricular Noncompaction: A Rare Case of Nonischemic Cardiomyopathy

**DOI:** 10.1155/2019/5637638

**Published:** 2019-08-08

**Authors:** Julia Tian, Asif Uddin, Philippe Akhrass

**Affiliations:** ^1^Department of Medicine, Northwell Health at Staten Island University Hospital, Staten Island, NY 10305, USA; ^2^Department of Electrophysiology, Northwell Health at Staten Island University Hospital, Staten Island, NY 10305, USA

## Abstract

Isolated left ventricular noncompaction (LVNC) is a rare form of cardiomyopathy that is characterized by deep intertrabecular recesses and abnormal trabeculations that can be observed on transthoracic echocardiogram (TTE) or cardiac MRI (CMR) studies. Our case describes a 41-year-old male who presented with exertional chest pain and was discovered to have significantly reduced left ventricular ejection fraction (LVEF) which was nonischemic in etiology as confirmed by cardiac catheterization. Subsequent evaluation with CMR imaging revealed noncompaction of the left ventricle. The patient received defibrillation and lifelong anticoagulation given his elevated risk of sudden cardiac death (SCD). This case highlights the importance of considering unconventional etiologies of cardiomyopathy when investigating new-onset heart failure as well as the necessity of life-saving measures such as anticoagulation and defibrillator implantation in view of arrhythmogenic structural heart diseases.

## 1. Introduction

Left ventricular noncompaction (LVNC) is an uncommon cardiomyopathy estimated to occur between 0.014 and 1.3 percent in the general population. It is characterized by deep intertrabecular recesses and abnormal trabeculations popularly believed to result from a failure of a phase of cardiac development during which the normal myocardium compacts inward. However, it can also involve abnormalities during ventricular wall formation or remodeling which can be transient or permanent [1, 2].

Clinical manifestations vary widely and can range from asymptomatic disease to symptoms of heart failure, left ventricular systolic dysfunction, thromboembolism, Wolff-Parkinson-White (WPW) syndrome, atrial and ventricular arrhythmias, bundle branch blocks, and sudden cardiac death. The most common manifestations are heart failure and ventricular systolic dysfunction.

There is currently no universally-accepted criteria for classifying and diagnosing LVNC. Depending on the organization, it may be classified as a genetic cardiomyopathy or as an unclassified cardiomyopathy [3, 4]. Diagnosis of LVNC can be made using imaging studies such as echocardiography, computed tomography, or CMR. Similar to the clinical criteria, no standard imaging criteria currently exist. The Jenni, Chin, and Stollberger criteria emphasize different components of the echocardiogram [5, 6]. A summary of all three would involve evidence of a two-layered structure (thin compacted epicardial layer and thick endocardial layer) with a noncompacted to compacted ratio of greater than 2 (greater than 2.3 on CMR), an epicardial-to-trabeculation trough to epicardial-to-trabeculation peak ratio of less than 0.5, and more than three trabeculations from the ventricular wall (not sensitive for CMR) [7, 8].

## 2. Case Presentation

**Figure 1:**
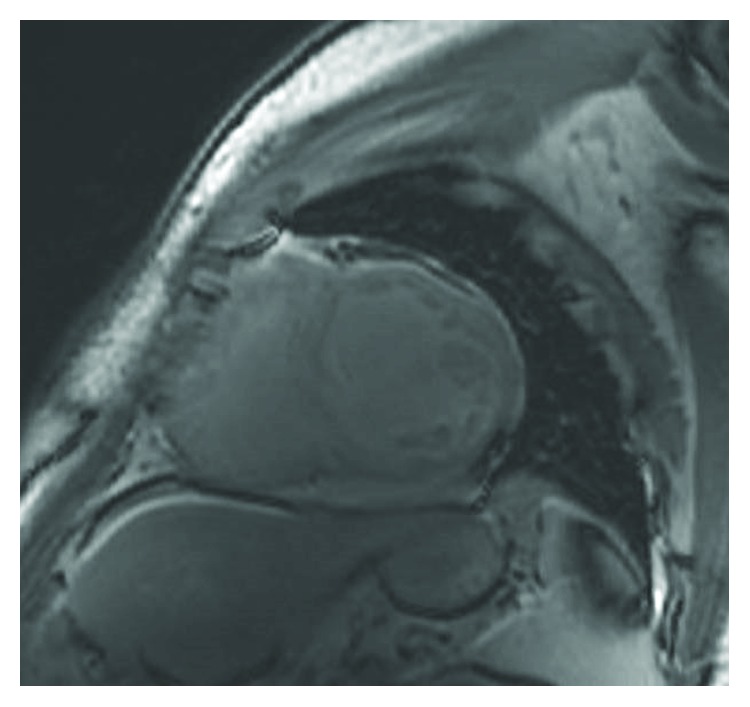
Short axis view of cardiac magnetic resonance (CMR) reveals a noncompact-to-compact myocardium ratio of greater than 2.3.

**Figure 2:**
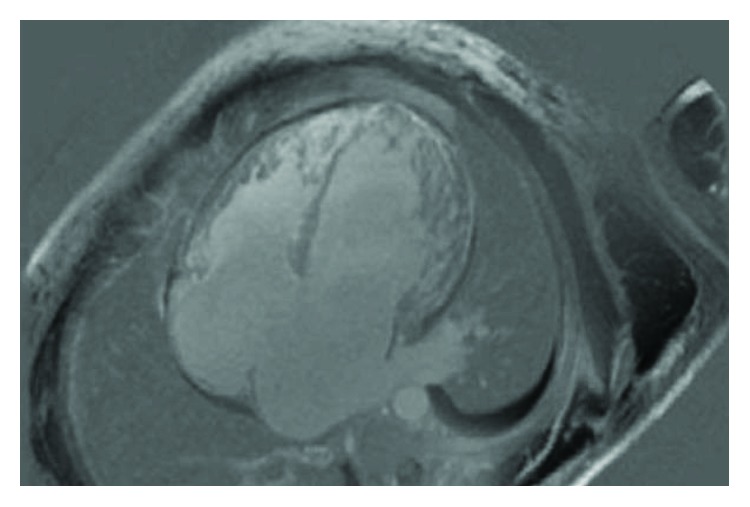
Four-chamber view of cardiac magnetic resonance (CMR) reveals dilation of all chambers of the heart.

The patient was a 41-year-old male with a past medical history of unspecified congenital heart disease, hypothyroidism, and hypogonadism (taking intramuscular testosterone injections twice weekly), who presented with a chief complaint of chest pain during exertion of 1-month duration. The pain was described as 10 out of 10 in intensity, burning with a band-like tightness in nature, and located in the epigastric area with radiation toward the substernal region. His initial cardiac enzymes were significant for a troponin level of 0.62 ng/mL. His B-type natriuretic peptide (BNP) level was 1,190. An electrocardiogram (ECG) revealed a sinus rhythm with right superior axis deviation, biatrial enlargement, and right ventricular hypertrophy. Further evaluation with a TTE revealed that the patient had a LVEF of 20-29% with a markedly dilated left atrium and left ventricle. Subsequently, a cardiac catheterization did not reveal evidence of obstructive coronary artery disease. Given the trivial history of congenital heart disease and nonischemic cardiomyopathy, a cardiac MRI was then conducted and revealed a noncompacted to compacted myocardium ratio of greater than 2.3 in the left ventricle, global hypokinesis with severely depressed left and right ventricle ejection fractions of 27% and 26%, biatrial enlargement, and no evidence of a left ventricular thrombus (Figures 1 and 2). His hospital course was complicated by an episode of nonsustained ventricular tachycardia, which given his LVNC and low ejection fraction, placed him at an elevated risk for SCD. The patient subsequently received an internal cardiac defibrillator (ICD) and was discharged home with aspirin, beta blocker, angiotensin-receptor blocker, and anticoagulation therapy with warfarin.

## 3. Case Discussion

In summary, LVNC is an uncommon and controversial cause of cardiomyopathy. It is becoming a popular topic in the field of cardiology due to its multiple possible etiologies, pathogenesis, diagnostic criteria, and clinical course. Due to its highly variable manifestations, the management of this condition should be individualized. Patients with evidence of heart failure should receive guideline-directed medical therapy (GDMT). Some literature supports the use of anticoagulation in patients with LVEF less than 40%, a history of atrial fibrillation, or a prior cardioembolic event [9]. All patients should also receive family and genetic counseling. As an uncommon disease with a multitude of life-threatening complications, it is imperative to perform a thorough cardiac evaluation in cases of new-onset heart failure with or without arrhythmia. In addition, further research and collaboration from major organizations are necessary to create a standardized criteria for the diagnosis and management of this disorder.
